# Oleic acid and derivatives affect human endothelial cell mitochondrial function and vasoactive mediator production

**DOI:** 10.1186/s12944-020-01296-6

**Published:** 2020-06-06

**Authors:** Virginia L. Bass, Joleen M. Soukup, Andrew J. Ghio, Michael C. Madden

**Affiliations:** 1 Department of Environmental Sciences and Engineering, Gillings School of Global Public Health, University of North Carolina at Chapel Hill, Chapel Hill, 27514 North Carolina USA; 2 Current Affiliation: RAI Services Company, Winston-Salem, NC USA; 3 Clinical Research Branch, Public Health and Integrated Toxicology Division, Center for Public Health and Environmental Assessment, ORD, U.S. Environmental Protection Agency, 104 Mason Farm Rd, Chapel Hill, NC 27514 USA

**Keywords:** Fatty acids, Endothelial cell, Vascular response, Particulate matter, Seahorse assay

## Abstract

**Background:**

Inhalation of common air pollutants such as diesel and biodiesel combustion products can induce vascular changes in humans which may contribute to increased mortality and morbidity associated with fine particulate matter exposures. Diesel, biodiesel, and other combustion byproducts contain fatty acid components capable of entering the body through particulate matter inhalation. Fatty acids can also be endogenously released into circulation following a systemic stress response to some inhaled pollutants such as ozone. When in the circulation, bioactive fatty acids may interact with cells lining the blood vessels, potentially inducing endothelial dysfunction. To examine whether fatty acids could potentially be involved in human vascular responses to air pollutants, we determined the effects of fatty acids and derivatives on important vascular cell functions.

**Methods:**

Human umbilical vein endothelial cells (HUVEC) were exposed in vitro to oleic acid (OA) or OA metabolites for 4-48 h. Cytotoxicity, vasodilator production (by ELISA measurement), mitochondrial function (using Sea Horse assays), and iron metabolism (inferred by ICP-OES measurements) were examined, with standard statistical testing (ANOVA, t-tests) employed.

**Results:**

Dose-dependent cytotoxicity was noted at 24 h, with 12-hydroxy OA more potent than OA. Mitochondrial stress testing showed that 12-hydroxy OA and OA induce mitochondrial dysfunction. Analysis of soluble mediator release from HUVEC showed a dose-dependent increase in prostaglandin F_2α_, a lipid involved in control of vascular tone, at 24 h (85% above controls) after OA-BSA exposure. RT-PCR analysis revealed OA did not induce changes in gene expression at noncytotoxic concentrations in exposed HUVEC, but 12-OH OA did alter ICAM and COX2 gene expression.

**Conclusions:**

Together, these data demonstrate that FA may be capable of inducing cytotoxic effects and altering expression of mediators of vascular function following inhalation exposure, and may be implicated in air pollutant-induced deaths and hospitalizations. (267 of max 350 words).

## Introduction

Exposure to ambient air pollution particulate matter (PM) is associated with increased adverse cardiovascular risk factors, including hypertension, atherosclerosis, and metabolic syndrome [1, 2]. Controlled PM exposure studies using diesel and biodiesel combustion emissions with human volunteers demonstrated an impaired endothelial-dependent vasodilatory response to agonists [3, 4], as one example of altered endothelial responses. One cellular mechanism attributed to PM-induced cellular effects is mitochondrial dysfunction [5, 6]. While transition metals associated with PM have been implicated in altered cell mitochondrial dysfunction and cytokine release [7, 8], other components of PM, such as fatty acids (FA), have not been as extensively studied. Non-esterified (i.e., free) FA, like oleic acid (OA), are bioactive and have been shown to impair the endothelium-dependent vasodilation mediator nitric oxide in cultured bovine pulmonary artery endothelial cells [9]. Elevation of circulating FA levels have been associated with endothelial dysfunction and endothelial nitric oxide system dysregulation in insulin-resistant and healthy subjects [10, 11]. Typical sources of circulating OA include diet and endogenous metabolism. Air pollution sources that contain a significant level of FA are generally derived from biomass combustion, such as cigarette smoke, diesel, biofuels [12], or meat cooking emissions [13]. Additionally, preparation of biofuels commonly involves the conversion of long chain FA from vegetable and animal fat triglycerides to mono-alkyl esters, though the conversion is generally incomplete. As a result, biofuels contain a mixture of lipid compounds, both alkyl-esterified (typically methyl) and unesterified [14]. In efforts to improve the cost and fuel efficiency of biodiesel, it has been suggested that source oils may be engineered with certain FA enrichments, including OA [12].

PM from the aforementioned pollution sources contain long chain FA on their surfaces that can enter the lung, and thereby translocate to the circulatory system, where they may induce vascular effects. It has been shown that the FA leukotriene B_4_ can translocate from the human airway lumen into the blood, demonstrating a potential route of exposure of the vasculature via inhalation [15]. Additionally, increases in circulating FA are known to occur by endogenous production following exposure to air pollutants like ozone, or cigarette smoke [16, 17]. Studies reporting vascular and pulmonary effects of OA suggest possible negative health effects from exposure through non-dietary routes [9–11, 18].

PM have been shown to alter the distribution of iron in the lung following inhalation [19]. This effect of altered iron localization has been observed with OA exposure in vitro*,* as well [20]. The possible mechanism behind the altered iron distribution may be due to the binding of iron to the FA due to the presence of the carboxy group [6, 21]. Little mechanistic work has addressed the potential contribution of iron chelation associated with particle exposures to biological effects [6, 20, 22–26].

We wanted to test whether the exposure of human endothelial cells to free OA could alter mitochondrial function responses and production of some mediators involved in the control of vascular tone. OA exposure may induce endothelial dysfunction which may be a potential mechanism implicated in some pollutant associated vascular effects.

## Materials and methods

### Materials

OA, conjugated OA-albumin from bovine serum (OA-BSA), methylated OA (me-OA), methyl-β-cyclodextrin (MBCD), oligomycin, carbonyl cyanide-4-(trifluoromethoxy) phenylhydrazone (FCCP), rotenone, antimycin A, glucose, sodium pyruvate, and ferric ammonium citrate (FAC) were purchased from Sigma-Aldrich (St. Louis, MO). 12-hydroxy OA (12-OH OA) was purchased from Cayman Chemical (Ann Arbor, MI). Two-hydroxy OA sodium salt (2-OH OA [Na^+^]) was purchased from Avanti Lipids (Alabaster, AL). 1-^14^C-oleic acid (58.2 mCi/mmol) was purchased from Perkin-Elmer (Boston, MA). Seahorse XF DMEM medium, XF24-well microplates, and XF Flux Paks were purchased from Agilent Technologies (Santa Clara, CA). Gibco L-glutamine was purchased from Thermo Fisher Scientific (Waltham, MA).

### Cell culture

Free OA, 12-OH OA, and me-OA were diluted in anhydrous EtOH to 700 mM, and then further diluted in cell media for use in cell exposures. Conjugated OA-BSA and 2-OH OA (Na^+^) were diluted directly in cell media. Equivalent vehicle controls were made with EtOH (< 0.14% EtOH final concentration) or BSA (< 33 mg BSA/ml) in EGM-2. MBCD was combined with OA in HBSS (Hanks Balanced Salt Solution; 6.37 mM OA, 38.2 mM MBCD) at a maximal binding ratio of ~ 1:6 OA:MBCD [27] and maximal MBCD concentration for water solubility, then sonicated and filter sterilized for dilution in dose response testing.

1-^14^C-OA uptake by HUVEC was determined by incubating cells with ~ 10^5^ CPM radioactive OA in 100 μM unlabeled OA for 4 h at 37 °C. OA was added with either BSA or ethanol (EtOH) vehicle. After 4 h or 24 h, cell media was removed, culture rinsed twice with PBS, and cells digested overnight in 1 M NaOH. Cellular uptake was assessed by measuring cell-associated ^14^C as a percentage of the total ^14^C recovered in cells plus media.

### FA preparations

Free OA, 12-OH OA, and me-OA were diluted in anhydrous EtOH to 700 mM, and then further diluted in cell media for use in cell exposures. Conjugated OA-BSA and 2-OH OA (Na^+^) were diluted directly in cell media. Equivalent vehicle controls were made with EtOH (< 0.14% EtOH final concentration) or BSA (< 33 mg BSA/ml) in EGM-2. MBCD was combined with OA in HBSS (6.37 mM OA, 38.2 mM MBCD) at a maximal binding ratio of ~ 1:6 OA:MBCD [27] and maximal MBCD concentration for water solubility, then sonicated and filter sterilized for dilution in dose response testing.

1-^14^C-OA uptake by HUVEC was determined by incubating cells with ~ 10^5^ CPM radioactive OA in 100 μM unlabeled OA for 4 h at 37 °C. OA was added with either BSA or EtOH vehicle. After 4 h or 24 h, cell media was removed, culture rinsed twice with PBS, and cells digested overnight in 1 M NaOH. Cellular uptake was assessed by measuring cell-associated ^14^C as a percentage of the total ^14^C recovered in cells plus media.

### Cell viability

To asses FA cytotoxicity, HUVEC were cultured in 96-well plates to 90–100% confluence, then exposed to EtOH, BSA, or MBCD solubilized OA, 12-OH OA, 2-OH OA (Na^+^), me-OA or vehicle controls, in quadruplicate, for 4, 24, or 48 h. Cell supernatants were collected immediately following exposure for use with the Promega Cytotox-96 cytotoxicity assay (Promega Corporation, Madison, WI) to measure supernatant lactate dehydrogenase (LDH) activity release. In some experiments, cell viability was visualized with standard light microscopy techniques by exclusion of trypan blue dye.

### Extracellular flux analysis

A whole cell mitochondrial stress test was performed (modified from Lavrich KS, Corteselli EM, Wages PA, Bromberg PA, Simmons SO, Gibbs-Flournoy EA and Samet JM [28]) using the Seahorse XF instrument (Agilent Technologies), which measures extracellular oxygen consumption rate (OCR). HUVEC were seeded at 40,000 cells per well in XF24 microplates 2 days prior to the assay and exposed for 24 h prior to assessment, with wells divided into blanks, vehicle control, 50, 100, and 250 μM OA, and 100 μM me-OA or 12-OH OA in EGM-2. Media was replaced with XF Cell Mito Assay Media (XF DMEM pH 7.4 with 10 mM glucose, 1 mM sodium pyruvate and 2 mM L-glutamine) immediately prior to the assay.

The Cell Mito Stress Test Assay was performed using injections of 1 μM oligomycin, 1.25 μM FCCP, and 0.5 μM rotenone, antimycin-A combination to measure OCR response. Adenosine triphosphate (ATP) production was measured using oligomycin, which inhibits ATP synthase. FCCP, a potent protonophore that makes the inner mitochondrial membrane permeable, was used to measure maximal respiration, and antimycin-a and rotenone, which inhibit complexes I and III to effectively halt mitochondrial respiration within the context of the assay, allow measurement of non-mitochondrial respiration. These injections also allow indirect calculation of additional bioenergetic parameters, including spare respiratory capacity, proton leak, and non-mitochondrial respiration.

### Soluble mediators

Cell supernatants were collected from 96-well plated HUVEC immediately following 24 h exposure to FA and were frozen until use in assays. Prostaglandin F2α (PGF2α) and endothelin-1 (ET-1) were measured by ELISA (Enzo Life Sciences, Farmingdale, NY and IBL-America, Minneapolis, MN, respectively) according to manufacture protocols. Dose-response measurements were compared as percent of control baseline concentration.

### Gene expression

Relative gene expression in HUVEC was measured using RT-PCR. Following 4 h OA-BSA dose-response exposures of HUVEC cultured on 12-well plates, RNA isolation was done using the Qiagen RNeasy kit (Valencia, CA) and quantified using a Nanodrop™ 1000 Spectrophotometer (Thermo Fisher Scientific, Waltham, MA). cDNA was generated as previously described [29]. Taqman pre-developed assay reagents from Applied Biosystems (Thermo Fisher Scientific, Waltham, MA): ET-1 (EDN1), endothelial nitric oxide synthase (NOS3) or custom-made: beta-actin (Actb), cyclooxygenase-2 (COX2), intercellular adhesion molecule 1 (ICAM1) (Supplement 1) were used for gene transcript detection by fluorogenic amplification of cDNA using the StepOnePlus detection system (Thermo Fisher Scientific, Waltham, MA). Dose response was compared using amplification cycle threshold (Ct) and each sample was normalized to Actb.

### Iron uptake

Confluent HUVEC cultured in 12-well plates were incubated with 100 μM FA (OA, 2-OH OA, or 12-OH OA) or vehicle in EGM-2 for 4 h. Media was aspirated and replaced with HBSS with or without 200 μM FAC for 1 h. following exposure, HBSS was removed and cells were rinsed with PBS. HUVEC were collected and hydrolyzed in 1 ml 3 N HCl and 10% trichloroacetic acid solution at 70 °C. Non-heme iron concentration in the supernatant was determined using inductively coupled plasma optical emission spectroscopy (ICPOES; Model Optima 4300D, PerkinElmer, Norwalk, CT) [19].

### Statistical analysis

Lactate Dehydrogenase (LDH) activity release relative to vehicle control was compared by multiple t-tests method with Holm-Sidak correction for multiple comparisons. Multiple comparisons by 2-way ANVOA with correction were used for soluble mediator release and iron-uptake assays. Gene expression fold change was compared relative to vehicle control groups with ∆∆Ct and significance was determined using a student’s t-test. All analyses were done with GraphPad Prism version 7 or 8. A *p*-value of < 0.05 was considered significant.

## Results

### Cell viability

**Fig. 1:**
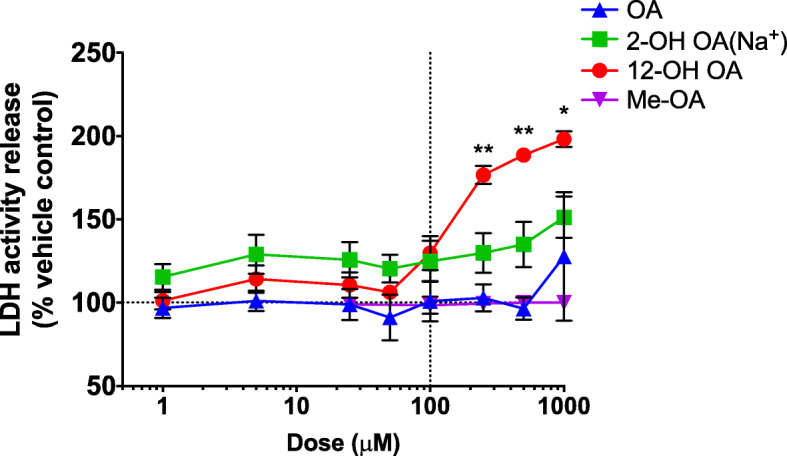
Comparison of cell viability in response to exposure of HUVEC to OA-EtOH, a hydroxy-metabolite, or me-OA as percent of the vehicle control response. Cell supernatant was collected after 24 h of exposure. Each value is the mean ± S.E.M. of 2–6 replicates. Comparison between OA and 12-OH OA groups done by multiple t-tests, * = *p* < 0.01, ** = *p* < 0.0001

Exposures of HUVEC cultures to FA showed that 12-OH OA significantly reduced cell viability at lower doses than OA after 24 h of exposure (Fig. 1). One hundred micromolar OA-EtOH and the OH- metabolites did not significantly increase the release of LDH in HUVEC. HUVEC exposed to me-OA at the same doses yielded no increase in LDH release.

A comparison of OA vehicles (Supplement 1, 2, 3) showed slightly greater cytotoxicity with a BSA vehicle, compared to EtOH, at 24 h. Time-course comparison of OA dose-response with an EtOH vehicle showed that cytotoxicity was also time-dependent between 4 and 48 h of exposure, with the cytotoxicity of SA relative to EtOH becoming significant at doses greater than 100 μM after 48 h of exposure. MBCD, a torus-shaped cyclic oligosaccharide with a hydrophobic cavity, was shown to induce significantly greater cytotoxicity as a vehicle for OA, starting at 500 μM and 24 h of exposure (Supplement 1, 2, 3).

Dose-response experiments showed that neither OA-EtOH nor OA-BSA induced significant cytotoxicity between 4 h and 24 h at 100 μM, and either was therefore suitable for use in assessing further sub-cytotoxic endothelial responses.

When measuring relative cellular uptake between EtOH and BSA vehicles, ^14^C-labeled OA exposures showed that 65% of labeled OA-EtOH became associated with HUVEC, versus only 8% of OA-BSA at 24 h (Supplement 1, 2, 3) suggesting different dosimetry of OA dependent on the vehicle.

### HUVEC mitochondrial response to FA

**Fig. 2:**
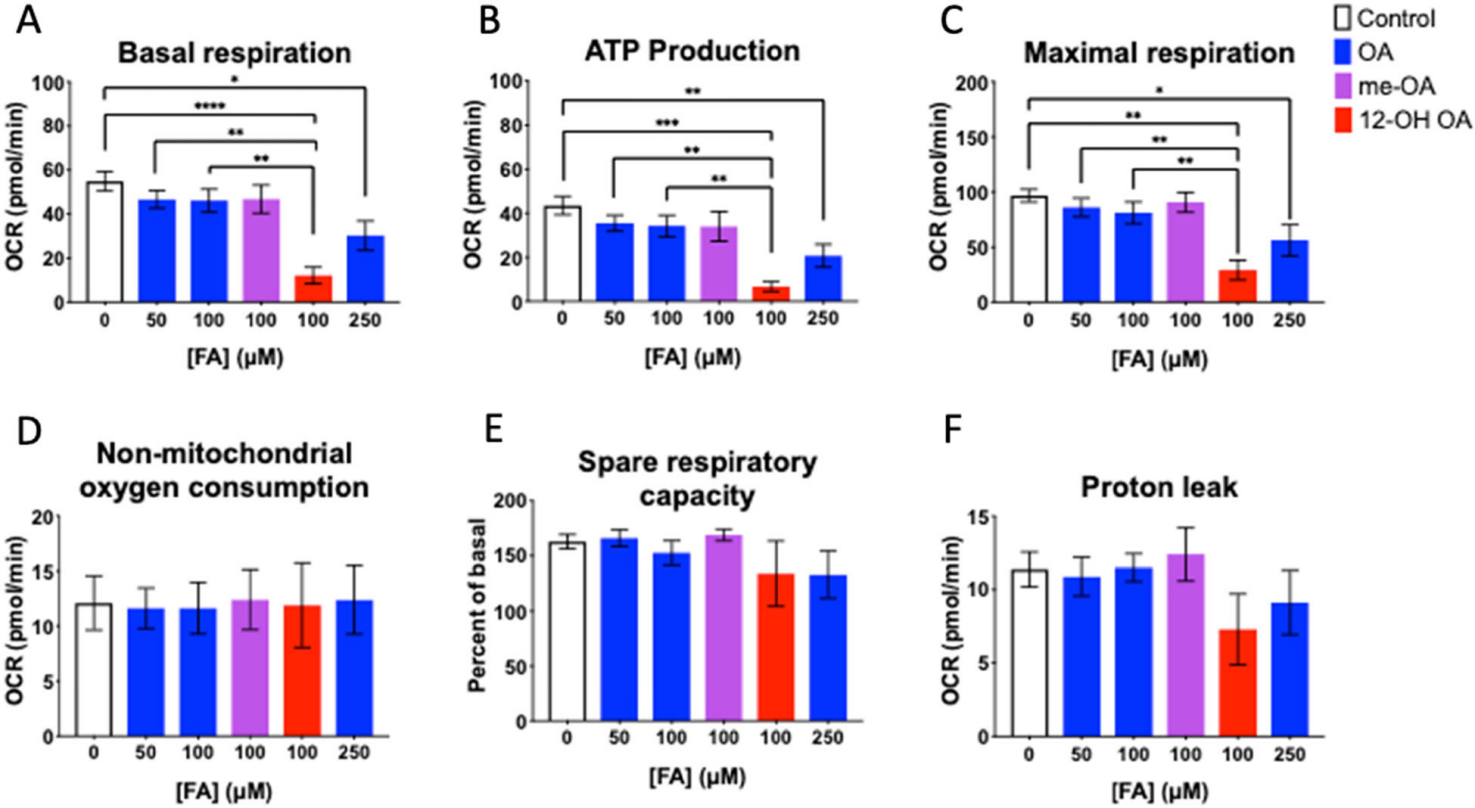
Measures of FA exposed HUVEC extracellular flux. Comparison of HUVEC oxygen consumption rate (OCR) measures, reflective of mitochondrial basal respiration (**a**), mitochondrial ATP Production (**b**), maximal mitochondrial respiration (**c**), non-mitochondrial respiration (**d**), mitochondrial spare respiratory capacity (**e**), and proton leak (**f**). All measures are reported as mean ± SEM, *n* = 5–8

HUVEC exposed to FA for 24 h prior to assessment by mitochondrial stress test showed indications of mitochondrial dysfunction at sub-cytotoxic doses of OA and 12-OH OA, but not me-OA. Basal mitochondrial respiration (Fig. 2a), expressed as the starting OCR, was observed to be decreased in HUVEC that had been dosed with either 100 μM 12-OH OA or 250 μM OA, relative to control. ATP production was also reduced in these treatment groups relative to control values (Fig. 2b). The addition of FCCP to effectively maximize mitochondrial respiration showed that cells treated with either 100 μM 12-OH OA or 250 μM OA had significantly reduced OCR relative to controls and lower OA doses (Fig. 2c). Following the addition of rotenone and antimycin-A to halt mitochondrial respiration, there was no difference seen in the relative rates of non-mitochondrial respiration measured (Fig. 2d). Spare respiratory capacity, calculated using basal and maximal respiration measures, and proton leak also did not vary significantly between treatment groups (Fig. 2e and f). Me-OA did not produce a significantly different response to any of these measures than either the control or 50 and 100 μM OA treated groups.

### Oleic acid-induced soluble mediator release

**Fig. 3:**
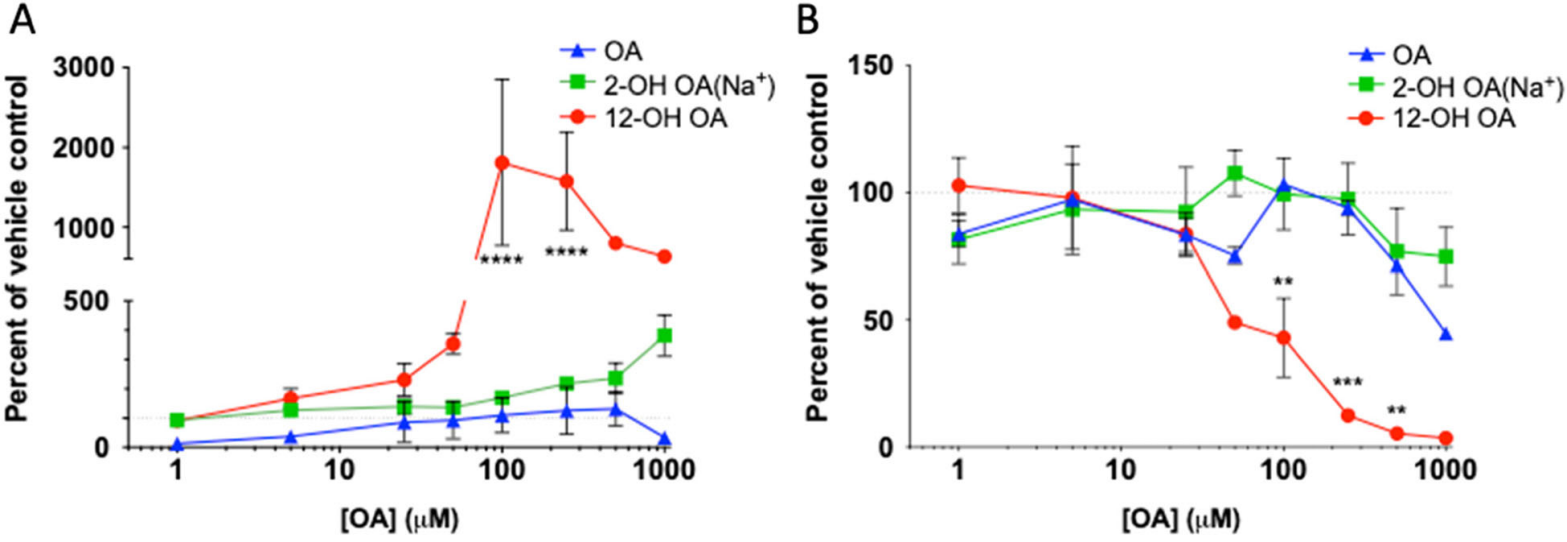
FA exposed HUVEC soluble mediator release. Comparison of soluble mediator release by HUVEC exposed to OA and its metabolites. Conditioned media was collected after 24 h exposure to OA-BSA, 2-OH OA (Na^+^), or 12-OH OA-EtOH or their equivalent vehicle controls. Mediators were measured by ELISA, as described in methods. **a** PGF_2α_ release. **b** ET-1 release. Each value is the concentration as a percentage of each group’s vehicle control, mean ± S.E. of 3 trials. Comparisons between groups by dose are done by two-way ANOVA. ** = *p* < 0.005, *** = *p* < 0.001, **** = *p* < 0.0001

Dose-response exposure of OA-BSA versus 2- and 12-OH metabolites showed significantly increased production of PGF_2α_, primarily in response to 12-OH OA at 100 μM and 250 μM compared with OA and control (Fig. 3a). Release of ET-1 was reduced by ~ 50% by 12-OH OA treatment at 100 μM (Fig. 3b), compared to OA and control, though all treatments caused a general decline in ET-1 production with increasing dose at 24 h exposure.

### Gene expression of endothelial signaling factors

OA exposure caused significant down-regulation of COX2 mRNA with 4 h 1000 μM exposure (Table 1), along with a general trend of down-regulation with increasing OA dose when compared to the control. 12-OH OA induced an almost 1-fold decrease in COX2 mRNA expression at 500 μM, as well as a significant decrease at 100 μM. No significant changes were seen in expression levels of endothelin-1 (EDN1) or endothelial nitric oxide synthase (NOS3) in response to either exposure. While OA did not change the relative abundance of ICAM1 mRNA, 12-OH OA caused a significant decrease in expression at both 100 μM and 500 μM, relative to the control.

**Table 1:** HUVEC gene expression changes

[OA] (μM)	0		100		250		500		1000	
Gene	*Mean ± SEM*	*n*	*Mean ± SEM*	*n*	*Mean ± SEM*	*n*	*Mean ± SEM*	*n*	*mean ± SEM*	*n*
EDN1	1.15 ± 0.57	2	*1.18 ± 0.48*	2	1.05 ± 0.39	2	*0.99 ± 0.46*	2	0.71 ± 0.18	2
COX2	1.02 ± 0.21	2	*0.95 ± 0.05*	2	0.8 ± 0.06	2	*0.67 ± 0.02*	2	0.21 ± 0.1 *	2
NOS3	1.01 ± 0.12	2	*1.15 ± 0.29*	2	1.14 ± 0.21	2	*1.3 ± 0.41*	2	0.79 ± 0.6	2
ICAM	1.01 ± 0.15	2	*1.04 ± 0.03*	2	1.1 ± 0.05	2	*1.18 ± 0.06*	2	0.39 ± 0.31	2
[12-OH OA] (μM)	**0**		**100**				**500**			
Gene	*Mean ± SEM*	*n*	*Mean ± SEM*	*n*			*Mean ± SEM*	*n*		
EDN1	1.05 ± 0.19	3	0.83 ± 0.35	3			0.98 ± 0.22	3		
COX2	1.21 ± 0.4	3	0.16 ± 0.08 *	3			0.05 ± 0.02 *	3		
NOS3	1.09 ± 0.26	3	1.58 ± 0.49	3			0.98 ± 0.36	3		
ICAM	1.46 ± 0.5	3	0.75 ± 0.22 *	3			0.22 ± 0.05 *	3		

Abbreviations: *Edn1* Endothelin-1, *Cox2* Cyclooxygenase-2, *Nos3* endothelial nitric oxide synthase, *Icam1* Intercellular Adhesion Molecule 1. Each value is the mean fold change ± S.E.M.. Mean expression values of markers are compared for significant exposure effects relative to vehicle-exposed group and normalized to beta actin (Actb) levels (* = *p* < 0.05)

### Oleic acid metabolite-induced cellular iron dysregulation

**Fig. 4:**
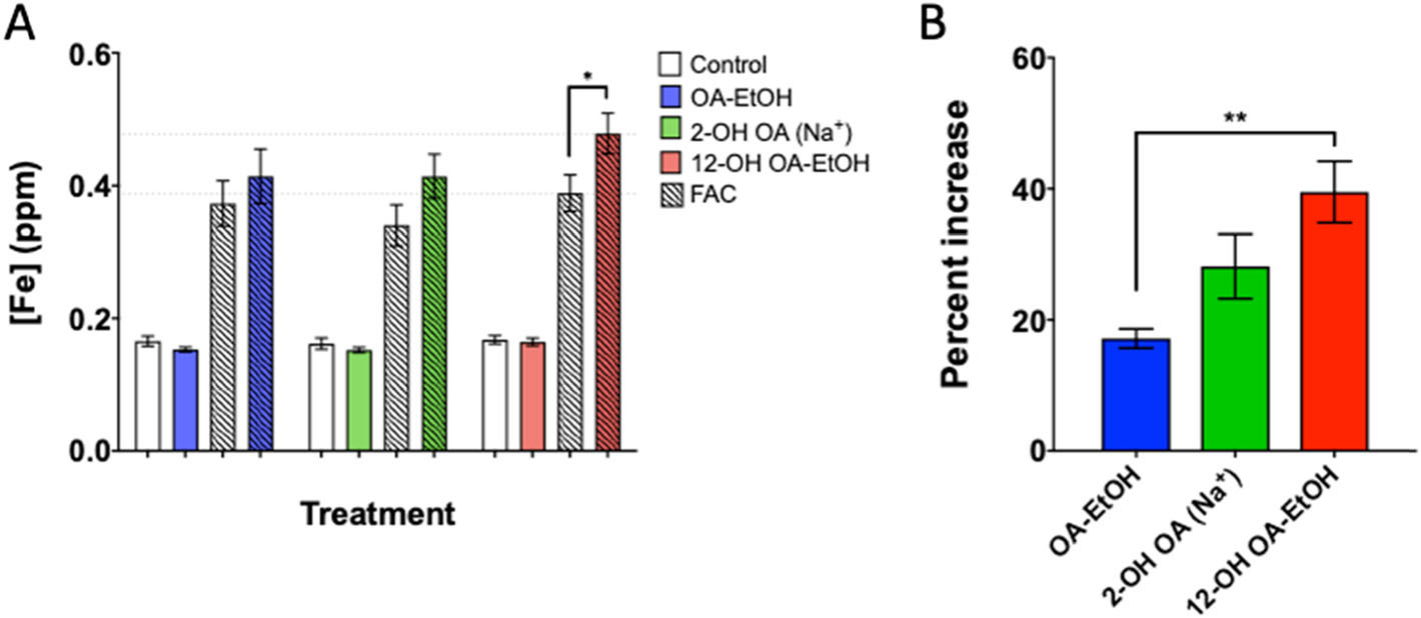
HUVEC iron uptake following incubation with FA. HUVEC in 12-well plates were incubated with 100 μM OA-EtOH, 2-OH OA (Na^+^), 12-OH OA-EtOH, or equivalent vehicle for 4 h in cell media prior to 1 h incubation with 200 μM FAC for 1 h in HBSS. Cells were processed and lysates analyzed by ICP-OES as described in methods. **a** Cellular iron concentration in control, FA, or FAC treated groups. **b** Percent increase in cellular iron concentration of HUVEC treated with both FA and FAC versus FAC alone. Each value is the mean ± S.E. of 3 trials

A 4 h exposure to OA, 2-OH OA, or 12-OH OA alone did not induce a change in iron content (Fig. 4a). When FA exposed cells were also incubated with FAC, both OA and 2-OH OA caused increases in cellular iron concentration relative to FAC alone (Fig. 4a), but only 12-OH OA significantly increased intracellular concentrations relative to vehicle control and FAC exposed HUVEC. The difference in iron content between FA exposed and unexposed cells post-FAC incubation, indicated as a percent increase, was 20% higher with 12-OH OA than OA (Fig. 4b).

## Discussion

Increased concentrations of circulating FA, as seen in patients with cardiovascular disease and diabetes mellitus, are considered a risk factor for CVD [30–32]. Environmental exposures that elevate circulating FA may also similarly pose a health risk. FA that have enter systemic circulation by deposition of PM and subsequent dissolution in the lung, or from endogenous production, have the potential to alter functions of endothelial cells that line and regulate the vasculature. Vascular effects of FA may link certain types of inhalation exposures to the adverse cardiovascular effects associated with air pollution, such as endothelial dysfunction, hypertension, and atherosclerosis. In a clinical study, plasma OA concentrations of 33.2μg/g [~ 117 μM] are reported for healthy subjects [33]. FA in rodent studies has been reported in the range of from 200 to 500 μM in serum [34, 35]. Studies reporting blood lipid composition in disease states consistently show increased levels of circulating FA, including OA. In a study of women with CVD, the percent of OA in the blood rose from 24.47 ± 6.18% in healthy women to 33.64 ± 7.88% in those with CVD [36]. Significant changes in other monounsaturated fatty acid (MUFA), like palmitic acid, and polyunsaturated fatty acids (PUFA) were also observed. MUFA were shown to comprise 40% of blood lipids in women with CVD, versus 30% in healthy women. Populations with preexisting CVD risk or with FA metabolism disorders might be more susceptible to FA-induced effects of air pollutant exposures due to normal compensation mechanisms. The wide ranges of free FA found in circulation of healthy subjects, versus the correlation of persistently high FA in diseased subjects, suggests that the general vascular environment may not be especially sensitive to intermittent high exposures. However, both i.t. instillation and i.v. injection of OA has been shown to induce pulmonary injury in vivo, and this organ system may be particularly sensitive to FA exposures. In the case of air pollutants, pulmonary vascular tissue exposure to OA may be more relevant in understanding the implications of FA contained on particulates. An animal inhalation model may provide useful insights into the question of effects mechanistically specific to this route of FA exposure.

The decrease in endothelial cell viability shown with OA exposure in this study reflected previous studies, which found cytotoxicity at doses above 200 μM at 24 h [37, 38]. Studies have also reported that me-OA is not bioactive in HUVEC and in general in other cell types [37, 39], however the effects of hydroxylated metabolites relative to those of OA are not as well understood. In the present study, we observed that OA and its metabolites alter endothelial cell mitochondrial function, vascular mediator production, and iron homeostasis at doses of 100-250 μM, at or below the threshold of cytotoxicity. Some fatty acids can induce apoptosis in HUVEC [40] though it is unclear how much this process contributes to OA-induced LDH increases compared with necrosis.

Endothelial mitochondria may, under some conditions, be involved primarily in cellular signaling, rather than meeting vascular tissue energy requirements [41, 42]. Changes in mitochondrial respiration produce downstream effects that impact the production and availability of NO and may translate to endothelial dysfunction in vivo. Experimental evidence supports a link between PM exposure and mitochondrial dysfunction, with metals components believed to be the active PM components [43]. In this study we further link FA to potential PM-induced mitochondrial changes and suggest a mechanism of OA-induced effects. FA have also been shown to elicit changes in reactive oxygen species production and the functioning of specific mitochondrial complexes [28, 34, 42, 44–46].

We show in vitro interactions of iron and FA in HUVEC, reflective of changes to iron homeostasis. Differences in the potency of OA versus hydroxylated (more potent) and methylated (less potent) forms of OA suggest that the addition of oxygen groups can increase potency of OA, whereas reduction of available oxygen groups may reduce potency of FA effects. Potency in this case may be due to an enhanced ability of FA to chelate iron. This study is the first to demonstrate this relationship with OA, and supports the hypothesis that OA might act on iron-containing complexes in the mitochondria, diverting iron away from them and toward other cellular pools.

Endothelial mitochondria, in addition to signal regulation, are shown to be critical in maintaining normal cellular iron homeostasis [44]. The same mechanisms that diminish mitochondrial respiration upon FA exposure, possibly resulting from disruption of iron-containing mitochondrial complexes, may reduce the ability of cells to regulate intracellular iron pools which could result in the increase in cellular iron in the presence of OA exposures in this study.

In this study, we have shown that incubation with OA species reduces oxygen consumption related to ATP production and general mitochondrial respiration. Additionally, OA and, to a greater degree, 12-OH OA upregulate the release of a potent vasoconstrictor, PGF_2α_, while reducing release of ET-1, which regulates both vasoconstriction and dilation. END1 gene expression was not altered at an earlier time (4 h) suggesting a nonoptimal sampling time for mRNA, and/or alterations in processing the mRNA and protein downstream. Both of these mediators are considered key early indicators of endothelial dysfunctions leading to cardiovascular disease pathogenesis [47, 48], and are involved in the pathways potentially impacted by mitochondrial dysfunction.

Expression of genes critical to endothelial signal transduction is relatively rapid and tightly controlled. In this study, the only significant change induced by OA exposure was in expression of COX2, an enzyme responsible for prostaglandin metabolism. A critical gene for control of endothelial mediated vasodilation, inducible nitric oxide synthase (iNOS), has been reported to non-inducible following initial transient expression in cultured HUVEC [49], so effects on this regulatory component are not apparent in this study, and would benefit from examination in a model with complete vascular tissue. Constitutive endothelial NOS did not show alteration in gene expression with fatty acid exposures (Table 1); additionally aortic rings did not have altered vasodilation after (1 h) incubation with 12-OH-OA in response to the NO donor sodium nitroprusside, but did with acetylcholine [50]. These findings suggest that NOS related pathways may not play a major role in 12-OH-OA effects on endothelial functions.

In summary, we have shown that OA and OH-OA exposed human endothelial cells have reduced mitochondrial function, exhibit altered signaling responses, and show changes in iron homeostasis. Combined, the findings in these experiments suggest a role of endothelial cell response that can relate ex vivo and in vivo effects previously observed to some of the epidemiological associations of ambient airborne PM pollutants and morbidity and mortality.

## Conclusions

The findings suggest that FAs, a component of combustion emissions PM, can induce in a vascular cell model biological effects that mimic responses observed upon in vivo inhalation of diesel and biodiesel. A potential target of cellular toxicity is cellular mitochondria with altered iron metabolism with more oxygenated FA having greater potency. FAs may play a potential role in altering human vascular responses which may induce increased morbidity and mortality upon PM and other air pollutant exposure.

## Supplementary information

**Additional file 1: Supplement 1.** Custom primer sequences used in RT-PCR.

**Additional file 2: Supplement 2.** LDH activity release is dose- and time-dependent.

**Additional file 3: Supplement 3.** OA cellular association differs with vehicle utilized.

## Data Availability

The dataset(s) supporting the conclusions of this article is (are) or will be available in the U.S. EPA Environmental Database Gateway repository at https://edg.epa.gov/metadata/catalog/main/home.page.

## Abbreviations

μM: Micromolar
Actb: β-actin
ANOVA: Analysis of variance
ATP: Adenosine triphosphate
BSA: Bovine serum albumin
COX2: Cyclooxygenase 2
cDNA: Complementary deoxyribonucleic acid
DMEM: Dulbecco’s modified eagle media
EGM-2: Endothelial (cell) growth medium-2
ELISA: Enzyme-linked immunosorbent assay
ET-1: Endothelin-1
EtOH: Ethanol
FA: Fatty acid
FAC: Ferric ammonium citrate
FCCP: Carbonyl cyanide-4-(trifluoromethoxy)phenylhydrazone
HUVEC: Human umbilical vascular endothelial cells
ICAM: Intracellular adhesion molecule
ICP-OES: Inductively coupled plasma - optical emission spectrometry
LDH: Lactate dehydrogenase
MBCD: Methyl- β-cyclodextrin
me-OA: Methylated oleic acid
mRNA: Messenger ribonucleic acid
NaOH: Sodium hydroxide
NOS3: Nitric oxide synthase 3
OA: Oleic acid
OCR: Oxygen consumption rate
PGF_2α_: Prostaglandin F_2α_
PM: Particulate matter
