# Supplementary material for: Oleic acid and derivatives affect human endothelial cell mitochondrial function and vasoactive mediator production
Source: Lipids Health Dis. 2020 Jun 6;19:128. doi: 10.1186/s12944-020-01296-6 (PMC7275404; doi:10.1186/s12944-020-01296-6)
Supplement: Supplementary file 1 — Additional file 1: Supplement 1. Custom primer sequences used in RT-PCR. [file 12944_2020_1296_MOESM1_ESM.docx]

## Supplement 1.

| **Gene** |  | **Sequence** |
| --- | --- | --- |
| ACTB | Forward | 5’-CCTGGCACCCAGCACAAT-3’; |
|  | Reverse | 5’-GCCGATCCACACGGAGTACT-3’; |
|  | Probe | 5’-ATCAAGATCATTGCTCCTCCTGAGCGC-3’ |
| COX2 | Forward | 5’-GAATCATTCACCAGGCAAATTG-3’ |
|  | Reverse | 5’-TCTGTACTGCGGGTGGAACA-3’ |
|  | Probe | 5’-TCCTACCACCAGCAACCCTGCCA |
| ICAM1 | Forward | 5’-GGAGGTCACCCGCAAGGT-3’ |
|  | Reverse | 5’-GCGGCTGCTACCACAGTGAT-3’ |
|  | Probe | 5’-ATGTGCTCTCCCCCCGGTATGAGATTG-3’ |

Supplement 1. Custom primer sequences used in RT-PCR.
