# Supplementary material for: Oleic acid and derivatives affect human endothelial cell mitochondrial function and vasoactive mediator production
Source: Lipids Health Dis. 2020 Jun 6;19:128. doi: 10.1186/s12944-020-01296-6 (PMC7275404; doi:10.1186/s12944-020-01296-6)
Supplement: Supplementary file 2 — Additional file 2: Supplement 2. LDH activity release is dose- and time-dependent. [file 12944_2020_1296_MOESM2_ESM.docx]

## Supplement 2.


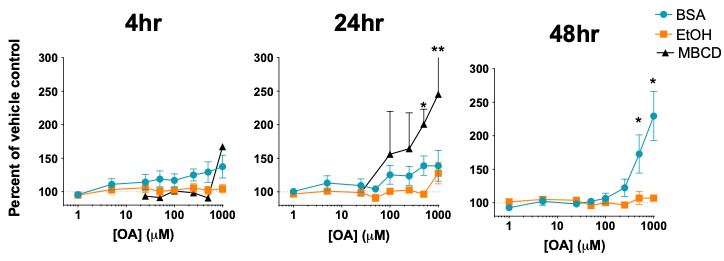


Supplement 2. LDH activity release is dose- and time-dependent, increasing between 1 and 1000uM and 4 to 48hr of exposure. HUVEC treated with OA-BSA showed greater increase in LDH activity release relative to EtOH. MBCD induced the greatest dose-responsive increase in LDH activity release, significant relative to EtOH at the highest two doses. LDH activity release was measured as percent of vehicle control. Values shown as mean ± SEM, 24hr compared by 2-way ANOVA, EtOH vs MBCD *=p<0.005, **=p<0.001; 48hr compared by multiple t tests, *=p<0.01.
