# Supplementary material for: Oleic acid and derivatives affect human endothelial cell mitochondrial function and vasoactive mediator production
Source: Lipids Health Dis. 2020 Jun 6;19:128. doi: 10.1186/s12944-020-01296-6 (PMC7275404; doi:10.1186/s12944-020-01296-6)
Supplement: Supplementary file 3 — Additional file 3: Supplement 3. OA cellular association differs with vehicle utilized. [file 12944_2020_1296_MOESM3_ESM.docx]

***Supplement 3***
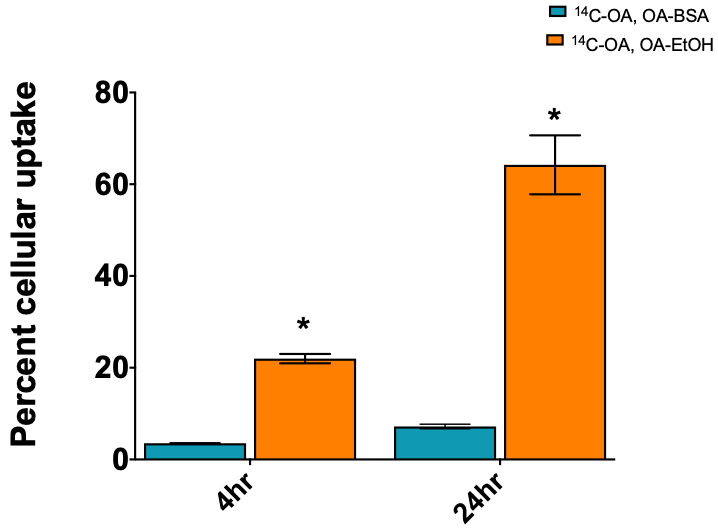


Supplement 3. OA cellular association differs with vehicle utilized. HUVEC treated with 100uM OA-BSA or OA-EtOH spiked with ^14^C labeled OA. Cellular uptake was measured as cellular fraction versus total recovered in solution after 4hr or 24hr incubation with HUVEC. Values shown are mean ± SEM, compared by a t-test. * = p< 0.0001
